# The Medical Aspects of the West Indian Islands

**Published:** 1902-05-31

**Authors:** Louis Vintras

**Affiliations:** Physician to the French Hospital.


### Hospital Clinics and Medical Progress.

### THE MEDICAL ASPECTS OF THE WEST INDIAN ISLANDS.

By Louis Vintras, M.D., B.Sc., Physician to the French Hospital.

Mountainous islands rich with tropical vegetation, separated from each other by large stretches of blue sea, where winter is unknown and the various seasons are all absorbed in one continuous summer, would tend, at first sight, to make the West Indies appear as a haven for all those who by constitution are weakly or whose frames have been debilitated by the touch of disease. Nor did I find on nearer acquaintance this first impression wholly incorrect; but here, as elsewhere, it is dangerous to jump at conclusions, and the indiscriminate dispatching of patients to the tropics is a practice against which we cannot too loudly protest.

Taking the West Indian Islands as a whole, and the differences between them are few, no climate could be better than that which is to be found among the hills. Here the air is pure, light breezes counteract the too fierce rays of the sun, and the temperature, though high, rarely becomes sultry. Unfortunately most of the towns, which were built for maritime and commercial purposes, lie on stretches of lowlands, often of alluvial, and mostly of marshy, formation, on the immediate foreshore of the islands; and in these towns the visitor must of necessity reside, for these islands, old English possessions that they are, have never been properly opened up, and in fact several are as yet in parts unexplored. The beautiful hills are either barren or covered with impenetrable vegetation, without the vestige of a village. The visitor must share with the population the mists and miasmas of the low-lying marshes, in view of those very hills where health awaits him, and which are for the present mostly impracticable for residential purposes. Hence the unenviable reputation which these islands have from time to time enjoyed.

Barbados is the first West Indian land that is reached, after leaving England, by one of the Royal Mail steamers, and has been looked upon for many years, by the inhabitants of the other islands, as a health resort. It is a coral island, and the highly porous nature of its subsoil renders it remarkably dry. It is well cultivated, hardly any portion of the land being left untilled; this being no small factor when considering it from the point of view of health. The air is extremely pure, and the Atlantic breezes sweep unchecked over the slightly undulated surface of the island, counteracting the excessive heat and making it the healthiest of the West Indies, and that in spite of the fact that it is one of the two or three most populated places on the face of the earth.

A run of over 700 miles to the westwards separates Barbados from Jamaica. Kingston is built on a broad marshy formation at the foot of the hills, and the fine harbour owes its unique appearance to a stretch of this same lowland thrown out as an arm and converting the broad estuary into a V-shaped channel. Arriving off Port Royal in the early morning, the only indication of Kingston is a heavy pall of mist hanging about the base of the mountains. This mist is known to the sailors as "the fever," and in their rough way they give utterance to a great scientific truth. Here, then, we have a typical West Indian town, built on marshy soil, with its sultry, moist, lowering heat, and above it the free slopes of the mountains, rising to shaggy crests, which cut a clear outline across the blue sky; below, the heavy, miasma-laden air; above, the light, pure atmosphere, the fresh breezes, and the promise of health. Something has been done of late to improve the town, and the establishment of the electric tramways, which now run in all directions, has necessitated the proper paving of the streets, most of which are lined with well-cemented gutters, where the water and refuse no longer stagnate; but there are many things still which are far from satisfactory. But we must not judge of Jamaica by Kingston alone; this picturesque island, as a whole, is in no way unhealthy; some of the smaller towns are delightfully situated, and present none of the unsanitary drawbacks of the capital; while outbreaks of yellow fever are now very rare.

Retracing our course eastwards, hugging the beautiful coast of Hayti, and passing Porto Rico, we reach the Leeward Islands, comprising Antigua, St. Kitts, Nevis, Montserrat, Dominica, and the two French possessions of Guadeloupe and Martinique. In this group we find many signs of that mysterious underground activity which plays so great a part in the geological revolutions of the globe. In Antigua and St. Kitts shocks of earthquake are frequent, and sulphur beds exist in both islands in a state of semi-activity. The mountains in these islands, with the zigzag markings of the old lava beds everywhere visible on their sides, and the crater-like formation of most of their summits, are so many volcanoes, none the less formidable for their unreliable quietude, as the recent terrible events have only too plainly shown. Antigua is flatter than the other islands, yet, strange to say, it is here that seismic movements of the ground are most frequent. With the exception of Dominica, the Leeward Islands are well cultivated, and possess excellent roads; and all, in point of view of health, leave little to be desired.

On either side of Dominica are the two French islands, of which Martinique was considered the most beautiful and the most healthy. The awful calamity with which it has just been visited has from all accounts ruined entirely this once fair island. St. Pierre, which, though not the capital, was the most important town, and of which now nothing remains but a heap of blackened ruins, was, though one of the oldest towns in the West Indies, remarkably healthy. It was built on the first slopes of Mount Pelée, and the incline which its streets thus possessed afforded an excellent drainage. I have visited St. Pierre several times, and have always found it free from the bad odours which are too often present in tropical towns.

The next group of islands includes St. Lucia, Grenada, and the ill-fated St. Vincent. St. Lucia is a military station, and there has been some talk of late years of making it the headquarters of the

West Indies; but while being exceedingly picturesque, it has many disadvantages. The interior is so wild that in parts it has not been explored; it possesses some valleys of extreme fertility and well cultivated, but the remainder is composed of unhealthy bush, and of all the West Indian islands it has the heaviest rainfall. Altogether I do not think it would prove so healthy a residence for the troops as Barbados, and I doubt very much how far it would be wise on the part of the War Office to effect the transfer. St. Vincent, now suffering from the havoc caused by the sudden activity of its highest volcano, the Soufrière, was in many respects one of the most beautiful and one of the most interesting of the West Indian islands, and from a medical point of view was second to none of them. Here no insalubrious foreshore, no low-lying valleys to gather the morning mists, no large agglomeration of inhabitants with the attendant evils. Excepting a rather heavy rainfall, everything seemed in favour of this island; the air is pure and light; the temperature varies between 79° and 90° Fahr., descending rarely below 70°; and the death rate was remarkably low. Yet it is this beautiful land which is now covered with volcanic dust, torn up by streams of lava, and burned by the continuous clouds of hot ashes which the Soufrière threw up for ten days in succession. I remember always with pleasure the impression of freshness and quietude I experienced on landing at Kingstown, the picturesque little town so happily spread across the broad valley rising gently from the open harbour, and which seemed so far remote from anything tragic. And yet the Soufrière was always looked upon with suspicion, and of all the volcanoes of the West Indies was thought by many to be the most likely to cause trouble; nor was its sympathetic activity, in conjunction with the eruption of Mount Pelée, a matter for much wonder to anyone familiar with these regions.

Entering the Gulf of Paria early in the morning by one of the Bocas, and steaming along the shores of Trinidad, one cannot but admire the beauty of the scene; but as Port of Spain is neared, the hills recede, leaving a stretch of lowland, on which stands the town, almost hidden under a mantle of mist. What I have said of Kingston, Jamaica, applies still more forcibly to Port of Spain—with this difference, however, that here the remainder of the island must bear with the town its share of the stigma of unhealthiness. It is useless for me to repeat the many drawbacks of this typical West Indian town, and all that need be said is that a place where the carrion-crow is protected by law to act as general scavenger is in a hopeless state of sanitary chaos. The heat in Port of Spain is most oppressive; it weighs on one's shoulders like a leaden mantle. The sea, the earth, the houses, seem to engender heat; and the mountains appear to be there only to throw back the heat of the town. The town is at times a perfect furnace. Trinidad is in no way a place for invalids, and should be studiously avoided by all who suffer from a sluggish circulation or from nervous prostration.

Such, then, are these beautiful islands, towards which the sympathy of the world has gone forth in their trouble, and which are, by a strange irony of Nature, at one and the same time so gifted and so fated.

